# Smoke-free home initiative in Bantul, Indonesia: Development and preliminary evaluation

**DOI:** 10.18332/tpc/113357

**Published:** 2019-11-15

**Authors:** Heni Trisnowati, Dian Kusuma, Abdillah Ahsan, Dwi E. Kurniasih, Retna S. Padmawati

**Affiliations:** 1 Division of Health Promotion Program, Public Health Department, Universitas Respati Yogyakarta, Indonesia; 2 Doctoral Program, Faculty of Medicine, Public Health and Nursing, Universitas Gadjah Mada, Yogyakarta, Indonesia; 3 Centre for Health Economics and Policy Innovations, Imperial College Business School, London, United Kingdom; 4 Faculty of Economy and Business, Universitas Indonesia, Depok, Indonesia; 5 Division of Health Policy and Administration Program, Public Health Department, Universitas Respati Yogyakarta, Indonesia; 6 Department of Health Behavior, Environment and Social Medicine, Faculty of Medicine, Public Health and Nursing, Universitas Gadjah Mada, Yogyakarta, Indonesia; 7 Center of Health Behavior and Promotion, Faculty of Medicine, Public Health and Nursing, Universitas Gadjah Mada, Yogyakarta, Indonesia

**Keywords:** secondhand smoke, smoke-free home, rural area, smoke-free environment, preliminary evaluation

## Abstract

**INTRODUCTION:**

Tobacco control policies in Indonesia are still limited. This study aims to describe the process of the implementation of the smoke-free home (SFH) program in rural areas in Indonesia and to conduct a preliminary evaluation of its implementation.

**METHODS:**

The development of SFH (or *Rumah Bebas Asap Rokok*) applies the theory of diffusion of innovation with the following stages: innovation, dissemination, adoption, implementation, and evaluation. The preliminary evaluation of the SFH program used an observational method combined with a cross-sectional survey. The population of this study was all houses in Karet hamlet, in Bantul district, Yogyakarta province with 378 houses as population, from which 196 houses were selected as sample using the proportional random sampling technique. Quantitative data analysis used multiple linear regression in Stata 15.1.

**RESULTS:**

SFH is a community-based tobacco control innovation program that began with a community declaration. Preliminary evaluation after one-year implementation showed that 55% and 45% of respondents were smokers and non-smokers, respectively. Among smokers, 95%, 78% and 56% reported not smoking near pregnant women, children, and non-smokers, respectively. Moreover, 52% of respondents reported having a front-door ashtray, and 46% reported guests not smoking; among non-smokers, the corresponding values were 56% and 60%.

**CONCLUSIONS:**

SFH implementation has an impact on the community’s smoking pattern. Awareness of smokers to protect women and children from secondhand smoke is very high. While the results are promising, more political and resource support is needed from the local and national policymakers to support SFH initiatives.

## INTRODUCTION

Tobacco use is the world’s leading cause of death that can be prevented^1^. One in ten deaths globally is caused by tobacco use^2^. If the trend continues, it would be responsible for more than 8 million deaths per year worldwide by 2030, of which 80% will occur in low- and middle-income countries (LMICs)^3^. In Indonesia, 56% of males and 2% of females, aged ≥10 years, were smokers in 2018, indicating very high exposure to secondhand smoke (SHS)^4^. The Global Adults Tobacco Survey (GATS) reported that 78% of adults were exposed to SHS at home in 2011^5^. Exposure to SHS increases the risk of cardiovascular diseases, respiratory diseases, and lung cancer, especially among the most vulnerable populations such as children and pregnant women^6-7^.

Tobacco control policies are still limited in Indonesia, the only country in the Asia Pacific region that has not ratified the Framework Convention on Tobacco Control (FCTC)^2^. While the tobacco industry is proliferating in the midst of minimal anti-smoking policies and regulations^8^, efforts are needed at the regional, district and village levels. The smoke-free home (SFH) initiative is a bottom-up effort to control smoking by involving the community directly with the primary goal of protecting non-smokers. Reducing the exposure to SHS, especially among women and children, is a significant public health challenge^9,10^. The SFH initiative has been developed in several countries, but the results vary. Experiences in high-income countries show that SFH is an effective means of reducing levels of cigarette consumption and increasing the likelihood of quitting smoking^11–13^. Experiences in LMICs such as India and Indonesia, especially in urban areas, indicate that the SFH initiative reduces smoking behaviour in homes and increases smokers’ readiness to stop smoking^14-16^. This study describes the efforts to develop an SFH initiative in rural settings in Bantul district, Indonesia, and provides a preliminary evaluation.

## METHODS

The development of SFH in Bantul (or *Rumah Bebas Asap Rokok*) applies the theory of diffusion of innovation with the following stages: innovation, dissemination, adoption, implementation, and evaluation. The evaluation used an observational method with a cross-sectional questionnaire survey. The population of this study was all houses in Karet hamlet, in Bantul district, Yogyakarta province with 378 houses as population, from which 196 houses were selected as sample using the proportional random sampling technique. Quantitative data analysis in Stata 15.1 employed multiple linear regression controlling for age, income, and having children. Ethical clearance was obtained from the ethics committee of the University of Respati Yogyakarta (No.236.3/UNRIYO/PL/XI/2018).

## RESULTS

Table 1 summarizes the development of the SFH initiative in Bantul (more details are given in the Supplementary file). In the first stage, *Innovation Development*, a need assessment was carried out to obtain preliminary data related to community smoking habits through surveys, interviews with community leaders, and stakeholders. Before the program, 90% of smokers were willing to smoke outside the home. Almost all residents agreed not to smoke inside the home. The next activity was the Village Community Deliberation with community leaders and health center officers to share the research findings and plan a follow-up in the form of an SFH program.

**Table 1:** Development stages of the SFH initiative, Indonesia

*Stage*	*Activity*	*Duration & number of villages*
Innovation development	Need assessment: survey smoking behaviour among communities in a village, interview with stakeholders Village Community Deliberation with community leaders and health centre staff	2 months (January–February 2017) 1 Village (378 households or 8 RT)
Dissemination	Regular meetings with a community group through socialization and education Installation of SFH stickers in front of residents' house installation of ‘ *cecekan* ’ (front-door ashtray)	2 months (March–May 2017) 1 Village
Adoption	The community has given a positive response to the SFH initiative andconducted declaration	1 month (May–June 2017) 1 Village
Implementation	House has front-door ashtray Guest not smoking at home Smoker does not smoke near family Smoker not smoking near children, pregnant women, and non-smokers Smokers do not smoke at community meetings	6 months or sooner (June 2017–November 2018)

RT: Rukun Tetangga or Neighbourhood Association.

The second stage, *Dissemination*, was to sensitise the targeted groups in the community on the SFH initiative. It was carried out through meetings with fathers, mothers, and youth groups. The SFH activities that were carried out included: 1) education about the dangers of smoking on health using movies and the measurements of blood pressure and blood sugar in the father meetings; 2) installation of stickers (‘Stub out cigarettes before entering the house’); 3) provide a ‘*cecekan*’ (frontdoor ashtray) outside the home throughout the community. The aim was for guests or families not to smoke inside the home.

The third stage, *Adoption*, was when the community responded positively, as shown by the SFH declaration. The declaration was signed by the hamlet head, youth organisations, and community leaders as representatives of the community. The declaration content included not smoking in the house; not smoking near pregnant women, children, and non-smokers; not smoking in community meetings, and installing a front-door ashtray. The declaration was made on 14 June 2017, one year before our preliminary evaluation (November 2018).

The fourth stage, *Implementation*, was when the community starts applying the content of the SFH declaration. The implementation depends on public awareness on the benefits of SFH and the support of available resources. During this stage, a preliminary evaluation was carried out to assess the implementation. Before the evaluation was conducted, an interview with community leaders showed that they were supportive and highly motivated to continue SFH towards realising a healthy and smoke-free village. A quote by the village leader: *‘… this village received the MDG Award in the field of nutrition in 2015, so we want to maintain that achievement with other efforts by implementing smoke-free houses, people who smoke should not be allowed in the house…’.*

One implementation challenge was that there were no clear sanctions for non-compliance with the contents of the SFH declaration. The implementation relied mainly on community awareness. Residents generally felt embarrassed if they were found to violate the declaration. Strengthening the sustainability of the program depends on the support of local leaders that are currently positive.

Smokers constituted 55% (n=108) of the study population. All the respondents were males with an average age of 44.7 years, and 85% (n=166) reported having children. Also, 51% (n=103) of respondents were in the first and second lower quintiles of income (i.e. more deprived), 18% in the third quintile, 15% in the 4th quintile and 13% in the highest income quintile.

Table 2 provides the behaviours related to smoking, one year after the SFH declaration. Results show that 55% and 45% of respondents were smokers and non-smokers, respectively. Among smokers, 95%, 78% and 56% reported not smoking near pregnant women, near children, or near non-smokers, respectively. Moreover, among smokers 52% and 46% reported having a front-door ashtray and guests not smoking; among non-smokers, the corresponding percentages were 56% and 60%. The mean differences of those indicators were mostly statistically significant, except for the house having a front-door ashtray.

**Table 2:** Proportion of respondents who engage in smoking related behaviors in Karet village, Bantul District, 2018

	*Overall (n=196)*		*Non-smokers (n=88)*		*Smokers (n=108)*		*Difference*	
	*Mean*	*SD*	*Mean*	*SD*	*Mean*	*SD*	*Mean*	*p*
	*[ 1 ]*		*[ 2 ]*		*[ 3 ]*		*[ 4 ]=[ 2-3 ]*	*[ 5 ]*
House has ‘ *Cecekan* ’ (front-door ashtray)	0.54	(0.50)	0.56	(0.50)	0.52	(0.50)	0.04	0.456
Guests not smoking	0.53	(0.50)	0.60	(0.49)	0.46	(0.50)	0.14	0.006
No family members smoking at gatherings	0.81	(0.40)	1.00	(0.00)	0.65	(0.48)	0.35	<0.001
Not smoking while near children	0.88	(0.33)	1.00	(0.00)	0.78	(0.42)	0.22	<0.001
Not smoking in community meetings	0.58	(0.49)	1.00	(0.00)	0.24	(0.43)	0.76	<0.001
Not smoking while near pregnant women	0.97	(0.16)	1.00	(0.00)	0.95	(0.21)	0.05	0.037
Not smoking near non-smoker	0.74	(0.44)	0.98	(0.15)	0.56	(0.50)	0.42	<0.001

SD: standard deviation, n: sample size. The mean and SD values are proportions.

## DISCUSSION

This research is different from previous work conducted in Yogyakarta city in terms of the focus on males of whom many are smokers and SFH activities. In Bantul, the activities included: showing educational videos on the dangers of smoking during the initial meeting with the fathers, screening of blood pressure and blood glucose for awareness, and installation of an ashtray in front of the house. The evaluation of the smoke-free home program was carried out after one year of program implementation to find out changes in the smoking pattern.

The proportion of smokers among adult males in Karet hamlet (55%) was similar to that in Indonesia (56%) in 2018^17^. This is because smoking is culturally acceptable in this community. However, people in the hamlet have the characteristic of being easy to receive new knowledge that is considered beneficial so that when the SFH initiative was introduced, the reception was welcoming. Even smokers supported the SFH initiative because they were aware of the dangers for their loved ones^12^. This is indicated by the relatively small (19%) proportion of smokers who smoked near family members in the hamlet compared, for instance, with Bantul district with 68%^17^. This is similar to the SFH initiative in Yogyakarta city that showed SFH implementation changed the community smoking pattern by an increase in the number of smokers who smoked outdoors, from 11% at the beginning of the intervention to 54% after the program^15^. SFH supports the national tobacco control, especially in Bantul, per Regent Regulation 18/2016 on Healthy Areas Smoke-Free that includes houses^18^.

### Limitations

This study has limitations, including that the preliminary evaluation was carried out simultaneously with the implementation so that success in quitting smoking is not causally related.

## CONCLUSIONS

While the preliminary evaluation of the SFH initiative shows promising results, more political and resource support are needed from the local and national policymakers. Further research (e.g. quasi-experimental impact evaluation) is needed to assess the impact of the SFH initiative on health behaviours and outcomes in the community.
