# Supplementary material for: Smoke-free home initiative in Bantul, Indonesia: Development and preliminary evaluation
Source: Tob Prev Cessat. 2019 Nov 15;5:40. doi: 10.18332/tpc/113357 (PMC7205119; doi:10.18332/tpc/113357)
Supplement: Supplementary file 1 [file TPC-5-40-s1.pdf]

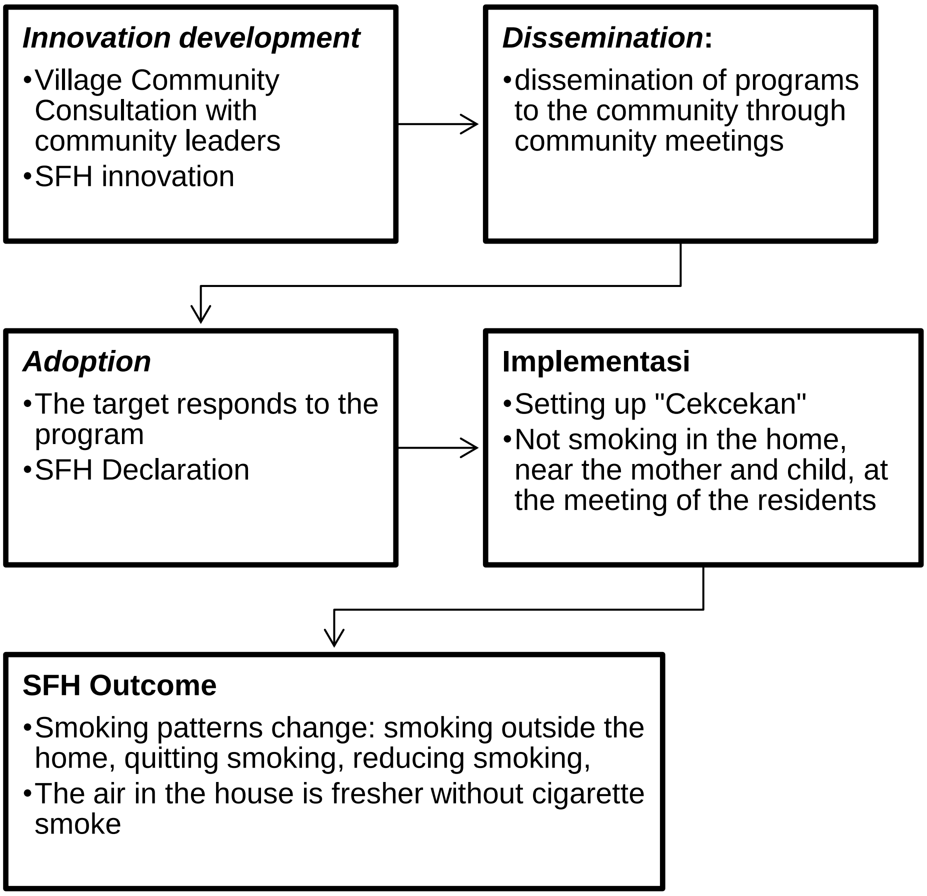

Figure 1. Stages of SFH development

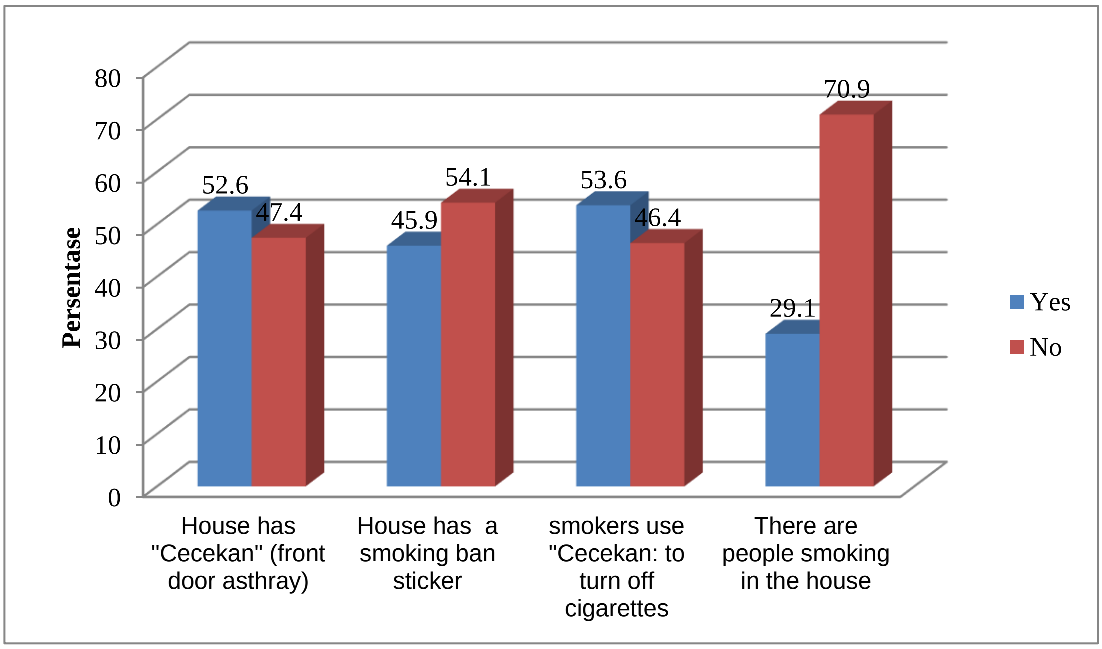

Figure 2. SFH observation results in Karet Village, Bantul District, Yogyakarta –Indonesia in 2018

© 2019 Trisnowati H.
